# A Question of Time

**Published:** 1914-07

**Authors:** 


### A Question of Time.

We ask our readers to bear in mind the obvious fact that it takes time to prepare matter for the printer, to set type, read proof, run the press, bind the forms, and wrap for mailing, also that a day each must be allowed for ink and glue to dry. As a matter of practical experience, we find that original articles, editorials, book reviews and as much as possible of other matter, must be sent to the printer on the first of the month before the date of issue. Brief personal notices, etc., can usually be inserted up to the 20th or 25th of the month. As we aim to mail each issue by the fifth of the month, it is obvious that it is not worth while to publish certain announcements, received in time for printing, but referring to meetings examinations, etc., to occur before the date of mailing. So far as possible, original articles will be published promptly, but this department is usually planned two or three months in advance, and articles are even printed in advance to facilitate measurement of space. We have not found it practicable to reserve space in advance. For example, just after the March issue was completely printed and awaiting binding, we had an urgent request to publish a certain article in it. The author was informed that he would be just barely in time for the April issue. That manuscript was received June 1, when the original articles for the July issue were already in the printer's hands. Such experiences, with minor exceptions as to dates, are the rule, not the exception. The editor admits that he is just as bad as anyone in this way. No practitioner, however good his intentions, can always fulfill his promises and expectations, with regard to the preparation of an article.

